# Reflex erection in the rat: reciprocal interplay between hemodynamic and somatic events

**DOI:** 10.1186/s12894-018-0352-5

**Published:** 2018-05-08

**Authors:** Alexander Andreev-Andrievskiy, Evgeniia Lagereva, Anfisa Popova

**Affiliations:** 1 Lomonosov Moscow State University, Biology faculty, 1-12 Leninskie gory, Moscow, 119234 Russia; 2 Institute for biomedical problems RAS, 76A Khoroshevskoe shosse, Moscow, 123007 Russia; 3 MSU Institute for mitoengeneering, LLC, 1-73A Leninskie gory, Moscow, 119234 Russia

**Keywords:** Reflex erection, Dorsal penile nerve, Intracavernous pressure, Bulbocavernosus muscle, Correlation

## Abstract

**Background:**

Penile erection is a complex reflex under spinal control and modulated by the brain. The hemodynamic events under autonomic control and the perineal muscles somatic activity are interconnected during the reflex erection at the spinal level, however if the afferent feedback on the corpus cavernosum pressure during an erection affects the somatic activity (perineal muscles contractions) and vice versa is not known. This study was aimed to test this hypothesis using a rat model.

**Methods:**

Intracavernous pressure (ICP) and bulbocavernosus (BC) muscle EMG were recorded during reflex erections elicited with dorsal penile nerve (DNP) electrical stimulation in anaesthetized acutely spinalized SD rats with surgically (bilateral cavernous nerve section, CnX, *n* = 8) and pharmacologically (trimetaphan infusion, TMPh, *n* = 8) abolished pressor response, or with surgically (bilateral section of the motor branch of the pudendal nerve, PnX, *n* = 7) and pharmacologically (1 mg/kg d-tubocurarine, *n* = 8) blocked perineal muscles contractions, or with interrupted afferent input from the penis (bilateral crush of the dorsal penile nerve, DPnX, *n* = 7). Control rats (*n* = 8) received no intervention.

**Results:**

Moderate positive correlations were found between net parameters of pressor and somatic activity during DNP-stimulation induced reflex erection in spinal rats, particularly the speed of pressor response development was positively correlated to EMG parameters. No changes of EMG activity were found in CnX rats, while the decrease of BC EMG in TMPh-treated males can be attributed to direct inhibitory action of TMPh on neuromuscular transmission. Pressor response latency was increased and ICP front slope decreased in dTK and PnX rats, indicating that perineal muscles contraction augment pressor response. DPN crush had little effect on ICP and EMG.

**Conclusion:**

Afferent input on the level of intracavernous pressure and the perineal muscles activity has minimal impact on, correspondingly, the somatic and the autonomic components of the reflex erection in spinal males, once the reflex has been initiated.

**Electronic supplementary material:**

The online version of this article (10.1186/s12894-018-0352-5) contains supplementary material, which is available to authorized users.

## Background

Penile erection is a complex reflex under spinal control and modulated by the brain [1, 2]. The sensory information from the penis is conveyed by the dorsal penile nerve, as the sensory branch of the pudendal nerve [3], to the spinal centers, displaying pattern generator and integrative properties [4–6]. The efferents include the autonomic proerectile parasympathetic neurons, the sympathetic neurons and the somatic afferents innervating the perineal muscles.

Reflex erection can be triggered by the descending input from the supraspinal sites [7] and the afferentation from the genitalia: tactile stimulation of the penis or electrical stimulation of the dorsal penile nerve. In rodents, when reflex erections are scored visually, the response includes penile tumescence, phasic penile glans engorgements and dorsoventral movements of the penis [8, 9]. Instrumental recordings during reflex erection in the rat [10] or mouse [11] reveal tonic pressure increase mediated by the parasympathetic neurons [12], superimposed with suprasistolic pressure peaks in the corpora cavernosum [10, 13] and spongiosum [14, 15]. The phasic pressure peaks arise concurrently with the perineal muscles EMG bursts and contractions [16], and denervation of the muscles abolishes these phasic events [17]. Apart from the crucial role the perineal muscles play in penile movements during copulation and glans erection, changes of reflex erection in paralyzed rats imply some auxiliary role of these muscles in initiation and/or maintaining the tonic pressure increase during the erection [12].

Persistence of reflex erections after thoracic spinal transection clearly illustrates self-sufficiency of the spinal level of erection control [8]. Tracing studies revealed profuse collaterals of the spinal preganglionic neurons within the spinal cord [5, 18–21], as well as ascending to supraspinal structures [3, 18, 22]. Numerous interneurons in the lumbar and sacral segments receive the afferent input from the penis [23, 24]. Glutamate [25, 26], acetylcholine [27], noradrenalin [28], serotonin [29–31], dopamine [32], GABA [33, 34], oxytocin [20, 35] and prostaglandins [36] are involved in erection control and provide ample neurochemical basis for complex regulation of the autonomic and somatic processes constituting an erection.

The hemodynamic and the somatic events during reflex erection are obviously time matched. The integration of these two components can be realized “directly” within the spinal cord, and there is also a possibility of “indirect” reciprocal influence of the afferent feedback on the level of somatic and autonomic activity, i.e. the afferent input on the level of intracavernous pressure could affect somatic activity and, vise versa, the perineal muscles proprioceptor feedback could modulate the parasympathetic or sympathetic neurons. It can be speculated, that this afferent interplay, if present, underlies the efficiency of physiotherapeutic treatments for erectile dysfunction based on stimulation/training of perineal muscles [37]. This study was aimed to investigate if the reciprocal afferent interplay between the autonomic and the somatic components of the reflex erectile response actually occurs. To this end we recorded intracavernous pressure and bulbocavernosus muscle EMG during reflex erection induced with DNP electrical stimulation in anaesthetized spinal rats with surgically or pharmacologically blocked hemodynamic or somatic component of the reflex response.

## Methods

### Animals and housing

Mature male SD rats weighing 500–600 g and 7–8 months of age were used in the study. Rats were purchased from the Institute of cytology and genetics SB RAS Animal resource center and were specific pathogen free. Males were housed in groups of 2–3 in semi-transparent cages (floor area 1500 cm^2^, Tecniplast, Italy) at 20–26 °C, 30–70% RH and 12-h light-dark cycle (lights on at 09:00). Standard rat chow (Assortiment agro, Russia) and reverse osmotic water were provided ad libitum. Wood chips were used for bedding; paper tissues and wooden sticks were offered to enrich the environment.

The study was approved by the Bioethics commission of MSU Institute for Mitoengeneering and performed in compliance with the European Convention for the Protection of Vertebrate Animals used for Experimental and Other Scientific Purposes.

### Experimental deign

Males were randomly divided into 6 experimental groups: 1) CnX animals (*n* = 8) that underwent bilateral ablation of the cavernous nerve, 2) TMPh (n = 8) group that received ganglionic blocker trimetophan; 3) PnX (*n* = 7) rats that were subject to bilateral sectioning of the pudendal nerve motor branch, 4) dTK (*n* = 8) rats that were paralyzed with d-tubocurarine, 5) DPnX (*n* = 8) rats had the dorsal penile nerve crush and 6) control rats (*n* = 8) that received no intervention. An on-line tool (https://www.graphpad.com/quickcalcs/randomize1/) was used for randomization. The sample size was calculated using G*Power (version 3.2) basing on the in-lab data on the parameters variability. After surgical preparation, two reflex erections were elicited in each animal before (baseline) and two more – after the experimental interventions were applied. The whole procedure was performed in urethane-anaesthetized animals. Intracavernous pressure and perineal muscles EMG were the primary outputs, with blood pressure and heart rate continuously monitored as indication of the animal’s general condition. Correction for baseline values collected in the same animal was used in order to minimize the inter-individual variability.

### Preparation

Experiments were performed with urethane-anesthetized rats (1.0–1.2 g/kg) using a procedure modified from Pescatori et al. [10]. Firstly, thoracic vertebral column was reached and cleared of the surrounding muscles. T9 vertebra was identified [38] and after laminectomy the spinal cord was transected at T8-T9 level. After the hemostasis was reached, the muscles and the skin were sutured in layers. Secondly, the right carotid and the left jugular vein were cannulated with PE50 tubing for direct blood pressure recording and fluid delivery correspondingly. The trachea was intubated to secure normal breezing or artificial ventilation if needed. Next, the penis was excised form the prepuce, cleared of the connective tissue and the left (chosen by convention) corpus cavernosum was cannulated with G23 injection needle on the ventro-lateral surface ≈5 mm distal to the crus for intracavernous pressure recording. The dorsal nerve of the penis was isolated bilaterally and placed on stainless steel electrodes. The bipolar G30 stainless steel electrodes spaced 3 mm apart (isolated for the whole length except for the bevel) were inserted into the left bulbocavernosus for EMG recording. All exposed tissues were covered with saline-soaked tissues to prevent dehydration and a small piece of cotton wool soaked in mineral oil was used to cover the DNP.

In addition to this general procedure, further preparation of the CnX, PnX and DPnX was performed. In CnX animals, access to the prostate was reached by bluntly separating the abdominal wall muscles, and the cavernous nerve was isolated bilaterally at the site of emergence from the major pelvic ganglion. The ligature was placed under both nerves and passed through a length of PE 100 tubing; after the baseline responses recording the ligature was pulled through the tubing so that the loop severed the nerve. In PnX rats, the motor branch of the pudendal nerve was accessed by gently separating the perineal muscles. Similarly to CnX rats, the ligature and a piece of tubing were used to form makeshift loop scissors around the nerve bilaterally. In DPnX rats the loose ligature knot was placed over the dorsal nerve of the penis distally to the stimulating electrodes and was tightened to crush the nerve after the baseline responses registration.

### Experimental protocol

Four reflex erections were elicited with DNP electrical stimulation with 4 V square pulses of 1 ms duration delivered at 5 Hz for 20 s; the intervals between the stimulations were 5–7 min. Analog BP, ICP and EMG signals were amplified, recorded at 5 kHz sampling rate and analyzed using PowerLab 8/30 and LabChart Pro. After the first two “baseline” responses the experimental interventions were applied. In CnX and PnX rats the cavernous nerves and the motor branches of the pudendal nerve correspondingly were severed; in DPnX animals the dorsal penile nerve was crushed. TMPh rats received 60 mg/kg trimetaphan as a bolus through the jugular catheter, followed by continuous infusion at 20 mg/kg/h. To maintain normal blood pressure in TMPh rats, angiotensin II was infused constantly at 15–75 mkg/kg/h rate (to the effect [39]). dTK rats were injected with 1 mg/kg d-tubocurarine into the lateral tail veins and immediately provided with artificial ventilation at 80 breaths per minute and 9–12 mmHg end tidal pressure (KTR-5 animal ventilator, Hugo Sachs Elektronik, Germany). After the treatments were applied, two more DPN stimulations were performed. The animals were euthanized prior to recovery from anesthesia via decapitation.

### Data handling and analysis

No a priori inclusion or exclusion criteria has been developed or used. Thus, all the collected data were included into the analysis.

The pressor response in the cavernous bodies was quantified on the basis of ICP/MAP ratio; to minimize the contribution of phasic pressure peaks, ICP traces were low-pass filtered (cut-off frequency 0.1 Hz) before division by MAP. Response amplitude (ICP A), area under the curve (ICP AUC), front slope (ICP IR, increase rate), latency (LP) and time to maximum (T_MAX_) were calculated. For EMG, cyclic analysis was performed to determine mean amplitude (EMG A), duration and frequency of each burst, AUC was calculated as a product of amplitude and duration. EMG activity was present mainly after the end of DNP electric stimulation; usually moderate to negligible EMG during the stimulation was distorted with stimulus artifacts and discarded from the analysis. The values for the two first responses were averaged to estimate the “baseline”, and averaging of the next two responses produced “treatment” values. The person preforming the original recordings analysis was blinded to the treatments.

Spearman’s correlations were calculated to estimate the relations between the ICP and EMG parameters for the pooled sample of “baseline” responses that were first averaged within the animal. For the “treatment” data, statistical analysis was performed using two-way ANOVA (factors “Group” and “Time”) on the values corrected for the group baseline means, followed with Sidak’s post-test. The correlations and differences were considered significant at *p* < 0.05. The data are presented as mean ± standard error of the mean.

## Results

### Reflex erectile response

**Fig. 1:**
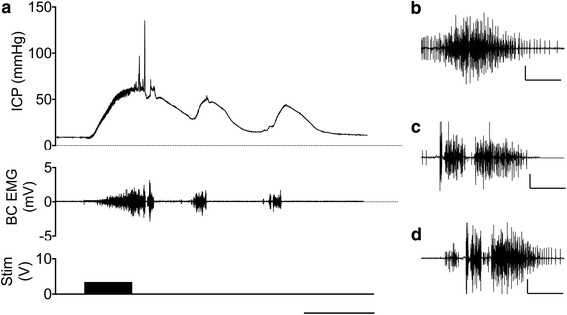
A representative reflex erection recording (**a**). Electrical stimulation of the dorsal penile nerve induced one to three (as in this recording) waves of tonic intracavernous pressure increase and bursts of bulbocavernosus muscle electromyogram. Concurrent with the perineal muscles contractions phasic peaks of intracavernous pressure occurred. Scale bar - 30 s. A “single” (**b**), “double” (**c**) and “serial” (**d**) bulbocavernosus EMG bursts. Scale bars - 2 mV/500 ms

For the “baseline” reflex responses, before the “treatments” were applied, the onset of the pressor component occurred on average 4.2 ± 0.3 s after the DNP stimulation start, ICP/MAP increased with an average slope of 2.06 ± 0.13 a.u./min and reached maximum amplitude of 0.651 ± 0.016 a.u. 29.7 ± 0.6 s after the response start. The first tonic pressure wave was often followed by a second one, and less frequently three waves were observed. The incidence of 1, 2 and 3-wave responses was 38.9, 55.6 and 5.6% correspondingly. The response lasted 83.7 ± 2.1 s and the AUC, as an integral characteristic of the erection intensity, was 28.51 ± 1.37 a.u. × s (Fig. 1a).

Bulbocavernosus EMG activity started simultaneously with the pressor response, but could be reliably quantified after the end of DNP stimulation only (Fig. 1a). EMG bursts, on average 9.4 ± 0.4 per response, were differentiated into three types. The most frequent (94.4 ± 1.4% of all bursts) “single” bursts (Fig. 1b) of varying shape and usually moderate amplitude, “double” bursts separated with 50–100 ms pause (Fig. 1c) and resembling “intromission” bursts [16] (5.0 ± 0.9%), and the most rare serial bursts (Fig. 1d), separated with 50–100 ms pauses in EMG activity (0.5 ± 0.5% of total number of EMG bursts per response). The amplitude of the dual and the serial bursts was approximately twice the single bursts amplitude. EMG bursts were associated with the front and the plateau of tonic pressure waves, at that, the dual and the serial bursts were always associated with the ICP plateau and produced phasic pressure peaks.

The response pattern, i.e. the number of tonic pressure waves and the pattern of EMG activity, was relatively stable within one animal, so that the correlation coefficients between the quantitative parameters of the first and the second “baseline” responses ranged ≈0.90–0.95.

### Correlations of hemodynamic and perineal muscles activity parameters

Parameters of the intracavernous pressor response and the bulbocavernosus muscle EMG were correlated during the reflexive erection (Table 1). Particularly, positive correlations were found between AUCs under the ICP/MAP and EMG curves, rate of ICP/MAP increase and EMG amplitude and EMG AUC, between ICP/MAP amplitude and EMG frequency, while latency of the ICP rise after the start of dorsal penile nerve stimulation was negatively correlated with the bulbocavernosus muscle electrical activity.

**Table 1:** Spearman’s correlations between ICP and EMG parameters

Spearman r(*n* = 46)		*ICP*				
		*AUC(a.u. × s)*	*A(a.u.)*	*IR(a.u./s)*	*LP(s)*	*Tmax(s)*
*EMG*	*AUC (mV×s)*	**0.354**	0.180	**0.370**	**−0.314**	−0.148
	*A (mV)*	0.121	0.181	**0.321**	−0.122	−0.201
	*F (Hz)*	−0.106	**0.303**	0.157	−0.119	0.010

Correlations significant at p < 0.05 are highlighted with **bold** text

### Changes of hemodynamic and somatic activity after surgical and pharmacological interventions

Blood pressure was unaffected by either of the treatments (F (5, 40) = 1.57, *p* = 0.1905) or time (F (1, 40) = 1.73, *p* = 0.1955). HR did not vary with the treatment (F (5, 40) = 0.65, *p* = 0.6618), but increased over the duration of the experiment (F (1, 40) = 38.47, *p* < 0.0001), particularly “after the treatment” HR was greater than the “baseline” in the control, trimetaphan and d-tubokurarine treated rats (*p* < 0.05, Sidak’s post test), but not in CnX, PnX and DPnX animals. No differences in BP or HR were found between any of the groups and the control rats either before or after the treatments (Additional file 1: Table S1).

In control rats, virtually no difference was found between baseline and “post-treatment” values of ICP/MAP or EMG parameters.

**Fig. 2:**
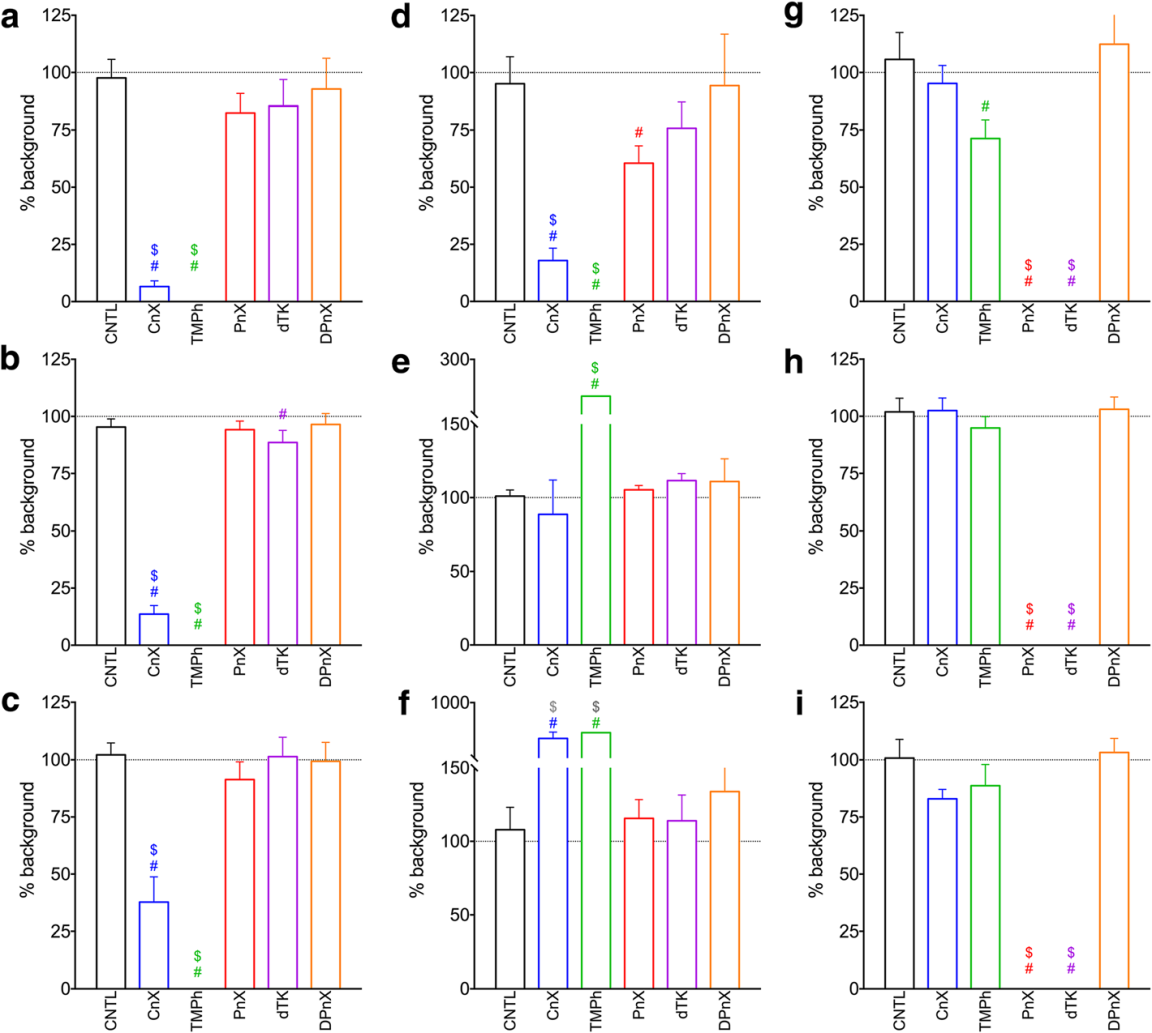
Erectile response (ICP/MAP) AUC (**a**), amplitude (**b**), duration (**c**), front slope (**d**), latency (**e**) and time to maximum (**f**), bulbocavernosus muscle EMG AUC (**g**), frequency (**h**) and duration (**i**) in control rats (CNTL, *n* = 8), animals with surgically (cavernous nerve crush, CnX, n = 8) or pharmacologically (trimetaphan administration, TMPh, *n* = 7) abolished erectile response, rats with surgically (motor branch of the pudendal nerve crush, PnX, n = 8) or pharmacologically (d-tubokurarine administration, dTK, n = 8) abolished perineal muscles activity, or after sensory dorsal penile nerve crush (DPnX, n = 7). The data are presented as percent of baseline (before the treatment) values. $ - *p* < 0.05 vs. control, # - *p* < 0.05 vs. baseline, Sidak’s post-test

Bilateral ablation of the cavernous nerve or ganglionic blockade with trimetaphan prevented ICP rise after dorsal penile nerve electrical stimulation (Fig. 2a-g, Additional file 1: Table S1). Trimetaphan administration was more effective, whereas some erectile response persisted in CnX animals due to incomplete sectioning of the diffuse cavernous nerve. No change of total bulbocavernosus muscle electrical activity was observed in CnX rats, while a 29 ± 8% decrease in EMG AUC followed TMPh administration, which was governed by reduced EMG average amplitude (− 15 ± 10%) and total EMG duration (− 11 ± 9%). Interestingly, EMG average amplitude was actually increased in CnX rats (11 ± 8%), and it was counterbalanced by shorter EMG total duration (− 17 ± 4%), resulting in EMG AUC little different from AUC before the nerve section (− 5 ± 8%). All EMG changes in CnX rats, however, did not reach statistical significance level (*p* > 0.05, Sidak’s post test).

Bilateral section of the pudendal nerve motor branch or d-tubokurarine administration resulted in complete loss of the bulbocavernosus muscle electrical activity and contractions after the DNP stimulation (Fig. 2g-i, Additional file 1: Table S1). Area under the ICP/MAP curve was somewhat lower in PnX (− 18 ± 8%) and dTK (− 15 ± 11%) animals (Fig. 2a), peak amplitude was decreased in dTK (− 11 ± 5%), but not PnX (− 6 ± 4%) rats (Fig. 2b), while pressor response duration was unaffected. ICP/MAP increase rate was diminished in PnX animals by 40 ± 8% (*p* < 0.05), and a comparable decrease (− 24 ± 11%) in curarized animals was not significant. Latency to ICP/MAP rise was longer in both PnX and dTK rats by ≈15% (*p* > 0.05, Sidak’s post-test), but the Tmax, measured from the response start, was unchanged.

In rats with dorsal penile nerve crush (located distally to the stimulating electrodes) no significant changes of either ICP/MAP or EMG parameters were found, however somewhat higher EMG AUC (+ 12 ± 16%) and longer latency of ICP/MAP rise (+ 13 ± 12%) should be noted.

## Discussion

Here we report existence of positive correlations between quantitative parameters of the reflex erection hemodynamic and somatic components in the spinal rat. Surgical or pharmacological blockade of the muscle activity resulted in a slower intracavernous pressure increase and in a generally smaller response, while electrical activity of the perineal muscles was not affected in rats with abolished pressor response. Interruption of the sensory input from the penis had little impact on the reflex erectile response in spinal rats.

### Reflex erectile response

Intracavernous pressor response in the DNP-stimulation induced reflex erection model we report here can be described as a tonic pressure wave(s) superimposed with phasic pressure peaks. It is reminiscent of the events during penile reflex tests in intact males [40], the apomorphine-induced erections [41], noncontact erections and, to a lesser extent, copulation [13] and can be mimicked with simultaneous stimulation of the cavernous nerve and the motor branch of the pudendal nerve [42]. Consistently with previous findings [10], the duration of ICP tonic increase was much longer in our experiments compared to penile reflexes tests with intact males, presumably due to disinhibition of the spinal centers and the intensive nerve stimulation used. In this regard it should be noted, that despite presumably the same neural pathways are activated during DPN electrical stimulation and tactile stimulation of the penile skin, they are not equivalent. At the very least, a mixed nature of dorsal penile nerve, that contains a portion of NO-ergic axons, that could directly induce intracavernous pressure increase upon electrical stimulation, should be mentioned [43].

In contrast to slower pressure events, the phasic pressure peaks are difficult to reliably measure, due to interference the cavernous tissue trabeculae create by mechanically obstructing the orifice of the cannula and the limitations of the catheter technique, when the long and relatively narrow fluid filled tubing can act as a low-pass filter. However, in case the peaks were registered (Fig. 1a), they did resemble those described previously in other erection models [13]. A similar pattern of pressor response is also found in the rat corpus spongiosum during reflex erections [14, 15]. The BS electrical activity in our study was also similar to the recordings made previously during reflex erections and mating [10, 16, 44]. Of particular interest, distinct EMG patterns that were recorded during intromissions [16], can be also observed in spinal males after DNP stimulation (Fig. 1b-d). Thus the responses we observed are in good accord with the existing data.

### Correlations of hemodynamic and perineal muscles activity parameters

Analysis of the quantitative parameters of ICP and BC EMG elicited with DNP stimulation revealed weak (≈0.3) but significant correlations between ICP and EMG responses, particularly, parameters that characterize the erection onset (latency and ICP increase rate) and EMG activity. Basing on the correlations found, several assumptions could be made: a) that the perineal muscles activity facilitates cavernous pressure increase, b) that the perineal muscles activity promotes greater erection intensity, or, alternatively, c) that the higher the intracavernous pressure and the faster it grows during the erection onset, the greater the muscle activity. The results of the transection and pharmacological experiments in this study indicate that the first two assumptions are true, while the latter is not.

### Pressor response inhibition has minimal impact on perineal muscles activity

After the pressor response was abolished via the cavernous nerve sectioning no changes of bulbocavernosus EMG were found, except for reduction of total EMG duration (insignificant), while in trimetaphan treated rats the EMG AUC was markedly reduced. The discrepancy between the denervation and ganglionic blockade results are explained by direct inhibitory action of trimetaphan on the neuromuscular transmission [45, 46], though not interfering with acetylcholine action directly [47]. Use of trimetaphan, rather than a more specific ganclionic blocker [48], could be considered a limitation of our study, however the differences in results obtained with TMPh and CnX rats underscore that abolished pressor response, and consequently, lack of the corresponding afferent input from the penis do not affect the perineal muscles motoneurons.

Ganglionic blockade leads to a marked drop in blood pressure, and ICP is directly dependent on the blood pressure level. To overcome the possible impact of low blood pressure on the outcomes of the study, we continuously infused angiotensin II to maintain arterial pressure within the normal range. It could be speculated that angiotensin itself could affect the pressor or somatic responses after DNP stimulation. However, unlike other systemic vasoconstrictors, particularly, adrenergic agonists, angiotensin displays pro-erectile properties [39], and low angiotensin concentrations have been shown to facilitate muscle contractions [49] through increased quantal content [50]. Thus, EMG inhibition found in trimetaphan treated rats can be attributed to direct effects of this ganglionic blocker on neuromuscular transmission and could not be governed by angiotensin.

The hemodynamic component of the reflex erection could affect the perineal muscles activity either “directly” through collaterals of the autonomic preganglionic neurons within the spinal cord or interneurons [5, 21, 23], or reflexively, as a result of afferent activity on the intracavernous pressure or penis distension. Our experiments were aimed to test the “reflex hypothesis”. Saline infusion into the penis can increase firing rate in the cavernous nerve, however, this activity persists after distal cavernous nerve crush [51], implying that the afferent feedback is conveyed by other routes, possibly, the dorsal penile nerve. The penile skin contains different types of sensory nerve endings, and electrophysiological studies reveal at least two types of slowly adapting neurons in the rat that could provide the afferent signal about penis engorgement. Interestingly, the reported adaptation period of tens of seconds in these slowly adapting units corresponds well with the erection duration [52]. The penile afferents innervate diverse regions in the spinal cord [19, 23, 53], and reach as far as the major pelvic ganglion [25] and could affect the motoneurons innervating the perineal muscles through pudendo-pudendal reflex [54] or other possible mechanisms. However, in our experiments rats with abolished pressor response had mostly unchanged perineal muscles EMG, thus the afferent feedback on the level of pressure increase does not seriously affect the bulbocavernosus motoneurons.

### Pressor response is decreased in absence of perineal muscles activity

After denervation of the perineal muscles or curarization the ICP rise was slower, the time to ICP/MAP maximum was somewhat longer, while the pressor response amplitude, and AUC, as an integral characteristic of the response intensity, were reduced. The pressor response changes in absence of the perineal muscles activity were relatively small, with the exception of the response onset latency that was increased by 40% in PnX and 25% in dTK rats. The longer pressor response latency we report here is in accord with findings of Giuliano at all made in a similar model with gallamine-paralyzed animals [12]. It should be mentioned, that the DPN stimulation was much longer in this study, while in our experiments a relatively brief electrostimulation was used to trigger the reflex response. Thus, our experiments provide more clear-cut data on the impact of perineal muscles contractions on the reflex erection pressor component. In intact males, denervation of the perineal muscles prevents glans erections in the penile reflexes tests [17] and impairs the ability of the male to reach an intromission during copulation [55].

The slower reflex pressor response in rats with blocked perineal muscles contractions can be explained by several possible mechanisms. One of them is a “mechanistic” explanation, namely that the muscles contractions facilitate penile veins occlusion or pump additional blood from penile crus and/or bulb and thus promote faster pressure increase. Alternatively, the action could be realized through diffusion of the motoneuron activity in the spinal cord or influence of the perineal muscle afferents. Little is known about the proprioreceptor feedback from the perineal muscles, however, in humans, spindles have been identified in the bulbocavernosus muscle [56] and stimulation of the perineal nerve elicits a reflex efferent activity [57]. Moreover, physiotherapeutic electrostimulation of the ischiocavernous muscle improves penile rigidity [37]. In the rat, contractions of the perineal muscles induce afferent firing in the cavernous nerve, that are abolished after neuromuscular blockade [51]. Thus, our results support the idea that the perineal muscles play an auxiliary role in initiating/maintaining erection, possibly by promoting compression of the penile veins and thus limiting the venous drainage, however, they do not rule out the other, “neurophysiological”, explanation.

In the reflex erection model used in our study, DPN crush, and thus interruption of the afferent feedback from the penis, had little impact on either pressor or somatic component of the reflex response. This result is in accord with previous report by Giuliano et al. in a similar model [42], where sectioning the dorsal penile nerve had little impact on the intracavernous pressure rise induced with DNP stimulation. Quite on the contrary, experiments with penile skin desensitization or sectioning of the pudendal nerve during copulation indicate that the sensation from the penile skin is critical to induce adequate somatic activity, for males with desensitized penises reached fewer intromissions than intact animals [55, 58]. Interestingly, male thrusting rate during a mount approximates ≈20 Hz in the rat [58], thus leaving ≈50 ms to initiate somatic events corresponding to an intromission, which is in good accord with the latencies of spinal components of pudendal and micturitions reflexes [51, 59]. In our experiments phasic tactile stimulation, like the one occurring during copulation, was presumably absent or minimal, but it can be speculated that slow adapting afferents were activated during the tonic intracavernous pressure waves. Thus, results of DPN crush experiments further support our conclusion that the feedback on the degree of penile tumescence does not affect the somatic perineal activity.

## Conclusion

In summary, our results indicate that once the reflex erection is initiated, there is little interplay between the pressor and the somatic components of the response, at least in the acutely spinalized rats. The somatic perineal muscles activity, however, augments the pressor response by mechanically accelerating the pressure increase.

## Additional file

Additional file 1 **Table S1.** Blood pressure, heart rate and reflexive erectile response parameters. (DOCX 22 kb)
