# Supplementary material for: Reflex erection in the rat: reciprocal interplay between hemodynamic and somatic events
Source: BMC Urol. 2018 May 8;18:36. doi: 10.1186/s12894-018-0352-5 (PMC5941648; doi:10.1186/s12894-018-0352-5)
Supplement: Supplementary file 1 — Table S1. Blood pressure, heart rate and reflexive erectile response parameters. (DOCX 22 kb) [file 12894_2018_352_MOESM1_ESM.docx]

**Table S1.**Blood pressure, heart rate and reflexive erectile response parameters

|  | **Control (n=8)** | **CnX (n=8)** | **TMPh (n=7)** | **PnX (n=8)** | **dTK (n=8)** | **DPnX (n=7)** |
| --- | --- | --- | --- | --- | --- | --- |
| *Blood pressure (mmHg)* | | | | | | |
| *Baseline* | 78.3±5.1 | 78.0±4.4 | 83.2±4.2 | 88.0±4.5 | 74.9±4.1 | 82.4±7.4 |
| *Treatment* | 78.0±3.9 | 81.4±3.3 | 94.4±1.9 | 86.4±4.1 | 72.9±4.3 | 80.5±7.4 |
| *Heart rate (bpm)* | | | | | | |
| *Baseline* | 245±15 | 248±19 | 228±18 | 250±14 | 215±11 | 258±15 |
| *Treatment* | 274±20 | 261±19 | 277±14 | 268±17 | 246±13 | 275±20 |
| *Area under ICP/MAP curve (a.u.*×s) | | | | | | |
| *Baseline* | 30.7±7.5 | 29.1±10.4 | 27.1±3.6 | 28.5±6.2 | 26.8±8.0 | 29.2±11.9 |
| *Treatment* | 30.0±7.0 | 1.9±2.0 | 0.0±0.0 | 23.5±6.8 | 22.9±8.7 | 27.1±10.4 |
| *ICP/MAP Amplitude (a.u.)* | | | | | | |
| *Baseline* | 0.69±0.04 | 0.66±0.04 | 0.69±0.02 | 0.66±0.03 | 0.64±0.03 | 0.62±0.05 |
| *Treatment* | 0.65±0.02 | 0.09±0.02 | 0.00±0.00 | 0.62±0.02 | 0.57±0.03 | 0.60±0.03 |
| *ICP/MAP pressor response duration (s)* | | | | | | |
| *Baseline* | 83.1±3.1 | 86.6±4.3 | 78.9±4.0 | 87.4±6.1 | 78.5±6.4 | 85.8±7.1 |
| *Treatment* | 84.9±4.3 | 32.7±9.6 | 0.0±0.0 | 79.9±6.8 | 79.6±6.6 | 85.3±7.1 |
| *ICP/MAP increase rate (a.u./min)* | | | | | | |
| *Baseline* | 2.13±0.24 | 2.30±0.42 | 2.14±0.30 | 1.87±0.25 | 2.04±0.23 | 1.76±0.28 |
| *Treatment* | 2.02±0.25 | 0.41±0.12 | 0.00±0.00 | 1.13±0.14 | 1.55±0.23 | 1.66±0.40 |
| *Latency of ICP/MAP rise (s)* | | | | | | |
| *Baseline* | 3.8±0.4 | 4.1±0.8 | 3.5±0.7 | 4.0±0.4 | 4.3±0.7 | 5.9±1.1 |
| *Treatment* | 4.1±0.6 | 19.7±3.7^1^ | 20.0±0.0^1^ | 4.6±0.5 | 4.9±0.8 | 7.9±2.1 |
| *Time to ICP/MAP peak (s)* | | | | | | |
| *Baseline* | 29.2±1.2 | 32.5±1.5 | 30.0±1.6 | 28.7±1.6 | 28.2±1.4 | 31.2±1.4 |
| *Treatment* | 29.5±1.2 | 28.8±7.6^2^ | 60.0±0.0^2^ | 30.2±0.8 | 31.5±1.3 | 34.6±4.8 |
| *Area under EMG (mV*×s) | | | | | | |
| *Baseline* | 28.6±10.7 | 36.3±9.6 | 26.0±6.9 | 31.6±10.8 | 28.2±8.2 | 28.0±6.7 |
| *Treatment* | 30.2±9.5 | 34.5±8.1 | 18.5±5.6 | 0.0±0.0 | 0.0±0.0 | 31.5±11.7 |
| *EMG amplitude (mV)* | | | | | | |
| *Baseline* | 2.05±0.19 | 2.63±0.19 | 1.84±0.12 | 2.10±0.29 | 2.22±0.13 | 1.78±0.18 |
| *Treatment* | 2.17±0.18 | 2.93±0.21 | 1.55±0.18 | 0.00±0.00 | 0.00±0.00 | 1.88±0.16 |
| *EMG frequency (Hz)* | | | | | | |
| *Baseline* | 303±11 | 303±12 | 294±8 | 288±16 | 317±10 | 302±13 |
| *Treatment* | 309±18 | 311±17 | 279±15 | 0±0 | 0±0 | 311±16 |
| *EMG duration (s)* | | | | | | |
| *Baseline* | 13.9±1.2 | 14.2±1.6 | 14.1±0.9 | 15.8±1.3 | 12.7±0.9 | 16.0±0.9 |
| *Treatment* | 14.0±1.1 | 11.8±0.6 | 12.5±1.3 | 0.0±0.0 | 0.0±0.0 | 16.5±1.0 |

^1^ – in case no ICP increase followed DNP stimulation, LP was attributed a value of 20 s

^2^ – in case no ICP increase followed DNP stimulation, Tmax was attributed a value of 60 s.
